# Return to sports activity after opening wedge high tibial osteotomy in patients aged 70 years and older

**DOI:** 10.1186/s13018-021-02718-6

**Published:** 2021-09-28

**Authors:** Akio Otoshi, Ken Kumagai, Shunsuke Yamada, Shuntaro Nejima, Takahiro Fujisawa, Kazuma Miyatake, Yutaka Inaba

**Affiliations:** Department of Orthopaedic Surgery, Graduate School of Medicine, Yokohama City University, 3-9 Fukuura, Kanazawa-ku, Yokohama, 236-0004 Japan

**Keywords:** Opening wedge high tibial osteotomy, Return to sports, Elderly patients

## Abstract

**Background:**

The purpose of this study was to evaluate return to sports (RTS) after opening wedge high tibial osteotomy (OWHTO) in elderly patients and associated factors affecting RTS.

**Methods:**

Seventy-four patients (mean age 68 years) who underwent OWHTO were enrolled. Clinical outcomes were evaluated using the Knee Society Score (KSS). Patients were asked regarding types of sports activities and their levels of participation within preoperative 1 year and postoperative 1 year. Levels of participation in sports and recreational activities were examined using the Tegner activity scale. The outcomes were compared between two age groups (≥ 70 years vs. < 70 years).

**Results:**

Of the 74 patients overall, 59 participated in at least one sport preoperatively, and 55 returned to sports postoperatively (RTS 93%). The KSS knee score and function score were significantly improved after surgery in both age groups (*P* < 0.05), but no significant differences were found between the age groups. The Tegner activity scales for ≥ 70 years and < 70 years were 2.9 ± 1.1 and 4.0 ± 1.9 preoperatively (*P* < 0.01) and 2.7 ± 1.2 and 3.3 ± 1.4 postoperatively (*P* = 0.16), respectively. RTS was reported by 24 of 25 (96.0%) in the age < 70 years group and 31 of 34 (91.2%) in the age ≥ 70 years group. Majority of age ≥ 70 years participated in low-impact sports preoperatively and returned to the same impact level postoperatively.

**Conclusions:**

The rate of RTS after OWHTO was high in patients aged 70 years and older with low-impact level. OWHTO is a preferred surgical option for elderly patients who desire RTS.

## Introduction

Osteoarthritis (OA) of the knee is a common disease in the aged population and the leading cause of restriction of activities of daily living (ADL). Since most countries have rising life expectancy and an aging population, extension of healthy life expectancy is a critical issue, and an improvement of quality of life, including enjoyment of sports activities, is often required.

High tibial osteotomy (HTO) is an established treatment option for OA of the knee. The procedure is performed to correct lower limb alignment and reduce mechanical force on the affected compartment. Proper overcorrection provides pain relief and subsequent improvement of knee function [1, 2]. The opening wedge HTO (OWHTO) has recently become commonly used, and good mid-term to long-term clinical outcomes have been demonstrated in patients over 70 years of age [3].

The advantage of HTO compared to knee replacement surgeries is that it is a joint-preserving procedure that is good for maintaining physical and sports activities [4]. Several studies reported a high rate of return to sports (RTS) activity after OWHTO [5–7]. However, most of the reports involved younger patients, and the rate of RTS in elderly patients is unknown. Furthermore, factors related to RTS after HTO have not been well-elucidated.

The purpose of this study was to evaluate RTS after OWHTO in elderly patients more than 70 years of age and identify factors related to RTS. It was hypothesized that the rate of RTS after OWHTO is high in patients aged 70 years and older.

## Materials and methods

### Patients

**Fig. 1:**
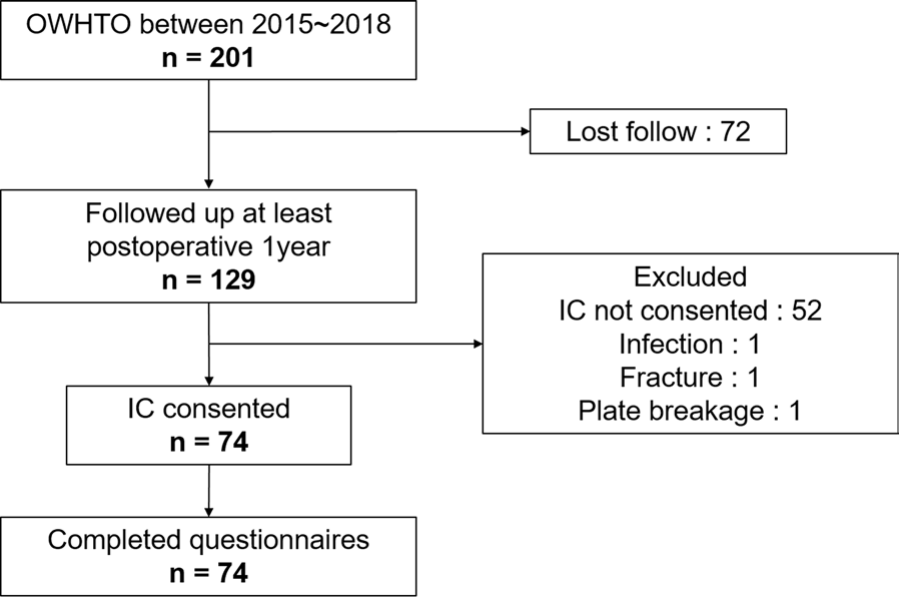
Inclusion flow diagram. OWHTO, opening wedge high tibial osteotomy. IC, informed consent

A total of 201 patients underwent OWHTO between 2015 and 2018. All patients underwent magnetic resonance imaging examination preoperatively to assess cartilage, meniscus, ligament, and subchondral bone in the affected knee joint. Inclusion criteria were painful osteoarthritis or osteonecrosis localized to the medial compartment of the knee. Exclusion criteria were patients with severe varus deformity (anatomical varus alignment > 5°), flexion contracture > 15°, or a history of inflammatory arthritis, joint infection, or immunosuppressive therapy. Following the surgery, 129 patients were followed-up appropriately for at least one year. Of these patients, 74 completed questionnaires (Fig. 1). The patients consisted of 55 female and 19 male patients, with mean age of 68.1 ± 8.1 years (median age of 70, range, 49–83 years). The mean follow-up period was 32.9 ± 12.9 months (range, 12–59 months). To evaluate the effect of age, outcomes were compared between two age groups (≥ 70 years vs. < 70 years). Demographic data are shown in Table 1. This study was approved by the institutional review board of Yokohama City University (#B190900037). Written, informed consent was obtained from all participants.

**Table 1:** Patients’ baseline characteristics

		Overall	< 70 years	≥ 70 years	*P value
Age, years		68.1 ± 8.1	61.3 ± 5.3	74.8 ± 3.7	
Number of patients		74	36	38	
Male		19	15	4	
Female		55	21	34	< 0.01
Body mass index, kg/m^2^		25.5 ± 4.6	26.1 ± 5.5	25.0 ± 3.4	0.34
Follow- up period, months		32.9 ± 12.9	33.8 ± 13.7	31.2 ± 12.6	0.18
OA, n		49	27	22	
^†^Grade	1	3	1	2	
	2	28	14	14	
	3	18	12	6	0.40
ON, n		32	14	18	
^‡^Stage	1	2	2	0	
	2	0	0	0	
	3	4	2	2	
	4	26	10	16	0.23

^*^ < 70 years vs ≥ 70 years

*OA* osteoarthritis, *ON* osteonecrosis

^†^OA grade modified from Ahlbach's classification

^‡^ON stage classification described by Koshino

### Surgical procedure and postoperative management

HTO was performed using biplanar opening-wedge technique with rigid plate fixation [3]. The amount of angular correction was planned preoperatively aiming to achieve tibiofemoral anatomical valgus of 10° in a one-leg standing radiograph postoperatively. The osteotomy gap was filled with two wedged blocks of β-TCP with 60% porosity (Osferion, Olympus Terumo Biomaterials. Corp., Tokyo, Japan) and fixed with TomoFix (DePuy Synthes, Zuchwil, Switzerland).

Patients started a postoperative rehabilitation program including isometric quadriceps and range-of-motion exercises the day after surgery. A non-weight-bearing regimen was prescribed for 1 week, followed by full weight-bearing exercise. Casts or supportive devices were not applied.

### Clinical and radiographic outcomes

Clinical outcomes were evaluated using the Knee Society Score (KSS), including the knee score and the function score. For radiographic assessment, a long-leg anteroposterior weight-bearing radiograph of the knee was taken 1 year after surgery. Limb alignment was expressed as the femorotibial angle (FTA), defined as the lateral angle between the femoral tibial axes [2], and the percentage of mechanical axis deviation (%MAD), defined as the ratio of the distance from the medial border of the proximal tibia to the mechanical axis of the lower limb to the width of the proximal tibia [8].

### Sports activity questionnaire

Patients were asked about types of sports activities and levels of participation within preoperative 1 year and postoperative 1 year. Levels of impact in sports activities were categorized into three groups: high-impact sports such as tennis, badminton, and running; intermediate-impact sports such as hill walking/hiking and climbing; and low-impact sports such as walking, gymnastic training, and golf [9]. If patients participated in two or more sports activities, the impact level was classified by the highest one. Levels of participation in sports and recreational activities were examined using the Tegner activity scale [10]. RTS was defined as preoperative and postoperative participation in one or more sports and recreational activities. Patients who took part in no sports preoperatively and started postoperatively were excluded from RTS. RTS levels were classified into the following three groups based on the preoperative and postoperative Tegner activity scales: higher level (Preop. < Postop.); same level (Preop. = Postop.); and lower level (Preop. > Postop.).

### Statistical analysis

Statistical analysis was carried out using SPSS ver. 26.0 (SPSS, Inc., Chicago, IL). The Mann–Whitney U test was used to compare measurements between the two groups. Pearson’s Chi-squared tests were used to test for significant differences of categorical data. Univariate analysis and multivariate logistic regression were used to identify factors related to the level of RTS. An adjusted *P* value < 0.05 was considered significant. A post hoc power analysis was performed for comparison of two age groups. Consequently, the statistical power was calculated to be 92% for a sample size of 36 and 38 in each group to detect a significant difference with an *α* level of 0.05.

## Results

### Clinical and radiographic outcomes

Clinical and radiographic outcomes are summarized in Table 2. The KSS knee score and function score were significantly improved after surgery overall and in both age groups (*P* < 0.05), but no significant differences were found between the ≥ 70 years and < 70 years groups. Standing FTA and %MAD were significantly changed after surgery in both age groups (*P* < 0.05), but there were no significant differences between the two age groups.

**Table 2:** Clinical and radiographic outcomes

		Overall	< 70 years	≥ 70 years	*P value
Knee score	Preop	57.3 ± 13.6	59.0 ± 13.0	57.2 ± 11.8	0.38
	Postop	83.9 ± 10.1	87.1 ± 8.3	82.8 ± 10.0	0.09
Function score	Preop	65.9 ± 11.1	66.0 ± 10.3	66.1 ± 11.8	0.69
	Postop	87.7 ± 12.2	89.9 ± 12.2	86.7 ± 12.5	0.20
sFTA	Preop	181.1 ± 2.4	181.2 ± 1.4	180.9 ± 2.4	0.50
	Postop	171.4 ± 2.8	171.8 ± 1.9	171.1 ± 2.9	0.59
%MAD	Preop	21.2 ± 11.7	20.2 ± 112.0	22.2 ± 11.4	0.39
	Postop	64.2 ± 10.8	66.0 ± 8.9	62.4 ± 12.1	0.26

^*^ < 70 years versus ≥ 70 years

*sFTA* standing femorotibial angle, *MAD* mechanical axis deviation

The values are given as mean ± standard deviation

### Return to sports

Types of sports activities are shown in Table 3. The number of low-impact sports increased postoperatively, whereas the number of intermediate-impact and high-impact sports decreased postoperatively. Comparisons of type of sports activities between age groups are summarized in Table 4. Of the overall 74 patients, 59 participated in at least one sport preoperatively, and 55 returned to sports postoperatively (RTS rate 93%). The number reporting RTS was 24 of 25 (96.0%) in the age < 70 years group and 31 of 34 (91.2%) in the age ≥ 70 years group. Seven patients who had not been involved in sports activities newly participated in sports activities after surgery. Overall, the rate of low-impact sports was greater and increased postoperatively. In the age ≥ 70 years group, one patient participated in high-impact sports (Softball) and three patients participated in intermediate-impact sports (Climbing). The majority of sports that participants in the age ≥ 70 years group were involved were low -impact sports preoperatively and postoperatively.

**Table 3:** Types of sports activities

Type of sports	Preoperative	Postoperative
**High-impact sports**		
Badminton	2	0
Running	2	1
Tennis	4	2
Baseball	1	1
Softball	2	2
Marathon	1	0
Volleyball	2	1
Table tennis	1	1
Total	15	8
**Intermediate-impact sports**		
Hill walking/Hiking	3	0
Climbing	3	3
Skiing	1	0
Total	7	3
**Low-impact sports**		
Walking	22	27
Gymnastic training	9	8
Swimming	4	6
Exercise/ Yoga	6	10
Golf	8	8
Gardening	2	3
Cycling	3	2
Tai Chi	0	1
Sports climbing	1	1
Total	55	66

The values are given as the number of patients

**Table 4:** Comparisons of return to sports activities and impact level between age groups

	Overall	< 70 years	≥ 70 years	*P value
	(*n* = 74)	(*n* = 36)	(*n* = 38)	
Preop. total participation in sports, *n* (%)	59 (79.7)	25 (69.4)	34 (89.5)	0.10
RTS	55 (93.2)	24 (96.0)	31 (91.2)	0.32
Postop. new participation in sports, *n*	7	5	2	
Postop. total participation in sports, *n*	62	29	33	
Preop. impact level, n High	12	11	1	
Intermediate	5	2	3	
Low	42	12	30	< 0.01
Postop. impact level, *n* high	7	6	1	
Intermediate	2	1	1	
Low	53	22	31	0.18

^*^ < 70 years vs ≥ 70 years

*RTS* return to sports

### Comparisons of RTS levels between age groups

Of the patients reporting RTS, 16 of 24 (66.7%) in the age < 70 years group and 24 of 31 (77.4%) in the age ≥ 70 years group could return to the same or higher level (Table 5). The Tegner activity scales decreased postoperatively in both age group; however, these were not significantly different. The preoperative Tegner activity scale was significantly lower in the age ≥ 70 years group than in the age < 70 years group (*P* < 0.01), but no significant difference was found in the postoperative score between the age groups.

**Table 5:** Comparison of level in return to sports between the age groups

		Overall	< 70 years	≥ 70 years	*P value
RTS, *n* (%) Higher level		1 (1.8)	0 (0)	1 (3.2)	
Same level		39 (70.9)	16 (66.7)	23 (74.2)	
Lower level		15 (27.3)	8 (33.3)	7 (22.6)	0.40
Tegner activity scale, mean ± SD	Preop	3.1 ± 1.8	4.0 ± 1.9	2.9 ± 1.1	< 0.01
	Postop	2.8 ± 1.5	3.3 ± 1.4	2.7 ± 1.2	0.16

^*^ < 70 years vs. ≥ 70 years

*RTS* return to sports

### Comparisons of outcomes between osteoarthritis and osteonecrosis

The outcomes were compared between osteoarthritis and osteonecrosis (Table 6). No significant differences were found in clinical outcomes, radiographic outcomes, RTS, and sports impact level between two disease entities.

**Table 6:** Comparisons of outcomes between osteoarthritis and osteonecrosis

		OA	ON	*P value
		(*n* = 42)	(*n* = 32)	
Age		68.7 ± 8.0	67.7 ± 8.1	0.81
Knee score	Preop	57.1 ± 12.8	57.5 ± 15.5	0.72
	Postop	83.9 ± 10.1	83.8 ± 10.5	0.86
Function score	Preop	67.6 ± 12.0	63.4 ± 9.5	0.15
	Postop	86.5 ± 13.0	89.4 ± 11.3	0.08
sFTA	Preop	181.8 ± 2.5	180.1 ± 2.0	0.24
	Postop	170.9 ± 3.1	172.0 ± 2.6	0.55
%MAD	Preop	18.9 ± 11.5	24.8 ± 11.4	0.89
	Postop	64.8 ± 10.7	63.3 ± 11.1	0.62
Tegner activity scale	Preop	3.1 ± 1.9	3.0 ± 1.8	0.54
	Postop	2.7 ± 1.5	2.6 ± 1.6	0.88
Preop. total participation in sports, *n* (%)		32 (76.2)	27 (84.4)	0.39
RTS, *n* (%)		29 (90.6)	26 (96.3)	0.21
Preop. impact level, *n*	High	7	5	
	Intermediate	4	1	
	Low	21	21	0.91
Postop. impact level, *n*	High	4	3	
	Intermediate	2	0	
	Low	29	24	0.8

*OA* osteoarthritis, *ON* osteonecrosis, *sFTA* standing femorotibial angle, *MAD* mechanical axis deviation, *RTS* return to sports

The values are given as mean ± standard deviation

### Factors related to the RTS level

To assess factors related to the RTS level, several variables were compared between RTS at the same or greater level and RTS at a lesser level by univariate analysis (Table 7). Significant differences were found in the preoperative knee score (*P* = 0.02) and the preoperative Tegner activity scale score (*P* < 0.01). Multivariate logistic regression analysis showed that only the preoperative Tegner activity scale score was related to RTS at the same or greater level, but age, sex, and BMI were not related (Table 8).

**Table 7:** Univariate analysis of factors related to the RTS level

Factors		Same or greater level	Lesser level	95% CI	*P* value
		(*n* = 41)	(*n* = 14)		
Age (years)		70.3 ± 6.9	68.1 ± 8.0	−0.012 to 0.014	0.89
Gender (male, %)		22	35.7	−0.311 to 0.091	0.27
Body mass index (kg/m^2^)		24.7 ± 3.4	24.8 ± 3.2	−0.017 to 0.028	0.63
Size of medial opening gap (mm)		12.2 ± 2.4	13.3 ± 3.3	−0.071 to 0.019	0.26
sFTA (degrees)	Preop	180.1 ± 2.3	181.1 ± 2.6	−0.049 to 0.036	0.75
	Postop	171.3 ± 2.7	171.2 ± 2.5	−0.031 to 0.034	0.92
Knee score	Preop	55.7 ± 11.6	63.1 ± 10.7	−0.017 to −0.002	0.02
	Postop	85.7 ± 9.5	82.5 ± 9.7	−0.012 to 0.017	0.72
Function score	Preop	65.3 ± 12.0	64.4 ± 10.4	−0.003 to 0.012	0.23
	Postop	90.1 ± 9.7	85.8 ± 16.0	−0.009 to 0.014	0.66
Preop. Tegner activity scale		3.5 ± 1.7	3.9 ± 1.4	−0.375 to −0.194	< 0.01

The values are given as mean ± standard deviation

**Table 8:** Multivariate logistic regression analysis of factors related to RTS at the same or greater level

Factors	95% CI	*P* value	Odds ratio
Age (years)	0.915 to 1.077	0.93	0.996
Gender (male, %)	0.255 to 3.906	0.92	0.934
Body mass index (kg/m^2^)	0.820 to 1.126	0.60	0.960
Preop. sFTA (degrees)	0.722 to 1.156	0.32	0.891
Preop. knee score	0.941 to 1.038	0.32	0.976
Preop. Tegner activity scale	0.439 to 1.032	0.04	0.663

## Discussion

The most important finding of the present study was that 91% of patients aged ≥ 70 years returned to sports activities, and 77% of them could perform postoperatively at the same or higher level compared to the preoperative level. These results were not significantly different from the younger age group. However, the ratio of high-impact sports participants was significantly lower in the age ≥ 70 years group than in the age < 70 years group. Factors related to RTS at the same or higher level were the preoperative knee score and the preoperative Tegner activity scale.

In general, ‘elderly’ is defined as a chronological age of 65 or more; however, there is considerable heterogeneity in current orthopedic research [11]. Age is an issue of debate as factor affecting clinical outcomes in HTO, and surgical indication of HTO is often limited to under 70 years [5, 7, 12]. Several studies focused the clinical outcomes in patients aged 70 years and older [3, 13]. Therefore, the present study referred to patients aged 70 years and older as ‘elderly.’

Recent studies have focused on RTS after HTO. A systematic review and meta-analysis of 33 studies involving 1914 patients with a mean age of 50.3 ± 9.9 years showed that the rate of RTS after OWHTO was 75.7% (range 55–100%) [14]. Another study investigating subjects with a mean age of 50 years demonstrated that the strongest prognostic factor for RTS was continued sports participation in the year before surgery (odds ratio 2.81; 95% CI 1.37–5.76) [12]. A high RTS rate after OWHTO has been reported in the relatively younger generation, and few reports have examined in detail the RTS rates in elderly persons. The present study showed that majority of patients aged 70 years and older participated in low-impact sports with high RTS rate, and age, sex, BMI, and knee alignment did not affect RTS rates.

One of the factors related to RTS in elderly persons is thought to be the extent of surgical invasion and recovery time. Improvements of surgical techniques and fixation devices in OWHTO have enabled early recovery with full-weight bearing, accelerated postoperative rehabilitation, and minimized muscle weakness [15, 16]. Early bone healing with a stimulatory device may also accelerate the weight bearing activities [17]. Accelerated rehabilitation protocols for OWHTO were introduced and lead to earlier improvement of the clinical results [15, 18]. In elderly patients, recent accelerated postoperative rehabilitation programs seems to work in favor of preventing muscle weakness and increasing RTS.

Types of sports seem to differ among age groups and may affect RTS after HTO. Younger people have a demand for returning to relatively higher impact sports, such as running, baseball, and tennis, whereas older people have a demand for lower impact sports, such as walking, gymnastic training, and golf. The present study demonstrated a higher rate of low-impact sports participants in the age ≥ 70 years group. Although the return to high -impact sports after OWHTO was high in the younger population [9], most patients return to sports activities with a trend toward performing lower-impact sports [19]. Thus, one of the reasons for the high rate of RTS in elderly persons may be the high rate of preoperative participation in low-impact sports. In addition, since the preoperative Tegner activity scale score in the age ≥ 70 years group was relatively low, it may not be affected by HTO surgery, and it is easy for the patients to return to the same level postoperatively.

Arthroplasty is an alternative treatment option for knee OA in elderly patients. The indication for unicompartmental knee arthroplasty (UKA) is similar to that for HTO, and a high RTS rate of 75–100% has been reported [20–22]. These rates are almost equivalent to the return rate in the present study. A recent systematic review indicated that UKA performed better than OWHTO in patients older than 50 years of age with compartmental knee OA secondary to frontal axis leg deformities [23]. However, there has been controversy over the studies of RTS directly comparing HTO and UKA. Jacquet et al. demonstrated quicker RTS with a higher rate of patients able to practice impact activity and better sports-related functional scores in HTO compared to UKA [24]. In contrast, Kim et al. reported that UKA had better short-term functional outcomes and return to recreational and sports activities than did HTO in patients with medial OA [20]. A systematic review and meta-analysis regarding RTS in elderly patients after UKA showed that higher return rates were observed for low-impact sports, whereas high-impact sports prevented a full return to activities [25]. Since there are risks of femoral component loosening and polyethylene wear in UKA [26–28], RTS after both surgical procedures needs to be assessed by long-term follow-up.

This study has several limitations. First, there were many cases that were lost to follow-up. More than half of the patients were excluded from this study, which may have caused selection bias. Second, the follow-up period was short. It is unclear whether patients who return to sports after surgery maintain sports activities at the same level for a long time. Third, this study was a retrospective investigation.

## Conclusions

The rate of RTS after OWHTO was high in patients aged 70 years and older with low-impact level. OWHTO is a preferred surgical option for elderly patients who desire RTS.

## Data Availability

The data and materials used and/or analyzed during the current study are not publicly available but available from the corresponding author on reasonable request.

## Abbreviations

OA: Osteoarthritis
ADL: Activities of daily living
OWHTO: Opening wedge high tibial osteotomy
RTS: Return to sports
KSS: Knee Society Score
FTA: Femorotibial angle
%MAD: Percentage of mechanical axis deviation
UKA: Unicompartmental knee arthroplasty
